# Comment on Published Article “Surgeon or Bot? The Risks of Using Artificial Intelligence in Surgical Journal Publications”

**DOI:** 10.1097/AS9.0000000000000357

**Published:** 2023-11-07

**Authors:** Hinpetch Daungsupawong, Viroj Wiwanitkit

**Affiliations:** From the * Private Academic Consultant, Phonhong, Lao People’s Democratic Republic; † Adjunct professor, Chandigarh University, India

We follow the topic on “Surgeon or Bot? The Risks of Using Artificial Intelligence in Surgical Journal Publications^1^”. ChatGPT, an AI system noted for its capacity to generate human-like prose, is said to have the potential to alter academia and pose ethical dilemmas in scientific literature production. The talk will center on the advantages and disadvantages of this technology in the surgical sector. In terms of benefits, ChatGPT has the ability to improve scientific writing by assisting in the generation of accurate and cohesive material. It can save time and effort for researchers by generating drafts, summarizing study findings, and assisting with literature reviews. This technology may potentially improve access to scientific knowledge by delivering automatic solutions to queries and increasing researcher collaboration.

However, there are hazards and ethical concerns involved with using ChatGPT in the surgical setting. One source of concern is the possibility of biased or erroneous information production because the AI model learns from current data, which may be restricted or skewed. If the AI is not adequately guided and supervised, there is a risk of disinformation or misinterpretation. Concerns have also been raised concerning the possibility of plagiarism or a lack of correct attribution when utilizing AI-generated text in scientific articles. The development of rules or frameworks for the responsible use of AI technologies such as ChatGPT in surgical literature writing could be a future approach in this area. This could involve creating transparency, disclosure, and verification criteria for AI-generated material.

Collaboration between AI developers, researchers, and publishers can assist in addressing ethical problems and ensure appropriate AI incorporation in scientific literature. More research is needed to investigate the possible influence of ChatGPT and other AI technologies in the surgical area in particular. Studies can look into the correctness and trustworthiness of AI-generated content, as well as the efficacy of AI aid in scientific writing and the ethical implications and concerns that come with its use. Furthermore, investigating the thoughts and opinions of surgeons, researchers, and publication ethics committees on the use of AI in surgical literature writing can provide useful insights for developing future recommendations and rules in this sector.

Modern algorithms and a substantial training set are essential for reducing bias and errors in chatbots.^2^ This is due to the possibility of problems when relying just on one significant data source. The use of chatbots raises ethical concerns since there may be unforeseen or detrimental effects. As AI language models develop, ethical criteria and limitations must be developed to prevent the spread of incorrect information and damaging ideas.

## ACKNOWLEDGMENTS

H.D. 50% ideas, writing, analyzing, and approval. V.W. 50% ideas, supervision, and approval.
